# Adult-onset hypothalamic hamartoma: origin of epilepsy?

**DOI:** 10.1186/s42494-023-00120-9

**Published:** 2023-04-28

**Authors:** Wenjie Han, Che Jiang, Zijuan Qi, Wei Xiang, Jian Lin, Youtian Zhou, Zhensheng Li, Bingmei Deng

**Affiliations:** Department of Neurology, General Hospital of Southern Theater Command, Guangzhou, 510010 China

**Keywords:** Hypothalamic hamartoma, Adult-onset, Gelastic seizure, Stereotactic radiofrequency thermocoagulation, Epileptogenic networks

## Abstract

**Background:**

Hypothalamic hamartoma (HH) is a congenital non-progressive lesion of hypothalamus during fetal development. Mass-like lesions in different anatomical locations often develop a variously disabling course presenting with cognitive decline, psychiatric symptoms, as well as multiple seizure types. As a rare disease, HH is relatively common in infants and children, but it is extremely rare in adults.

**Case presentation:**

We reported a case of adult-onset hypothalamic hamartoma, and summarized and analyzed relevant reports and studies of HH worldwide. The patient had clinical manifestations characterized by multiple seizure forms. After stereotactic radiofrequency thermocoagulation and drug treatment, the condition was effectively controlled. The patient was followed up till October 2022, with no recurrence of seizures.

**Conclusions:**

Epilepsy caused by HH can resemble that of temporal lobe seizures, as HH forms a complex epileptogenic network with other regions of the brain through anatomical and functional connections. Early treatment of HH can provide better control of the symptoms of epilepsy, and patients with longer disease courses may have more complications.

## Background

Hypothalamic hamartoma (HH) is an ectopic, non-neoplastic nerve tissue containing normal neurons and glial cells (oligodendrocytes and astrocytes) [1], which generally originates from the third ventricular floor, tuber cinereum, or mammillary body [2, 3]. HH is extremely rare in clinic, with an incidence of one per 50,000–100,000 [4]. HH can cause neurological and endocrine symptoms, including gelastic seizure (GS), dacrystic seizure, and other types of epilepsy. It can also lead to precocious puberty, abnormal behavior, and progressive cognitive deterioration [1]. Although HH is common in infants and children, it is extremely rare in adults [5, 6]. Nguyen reviewed 277 HH patients published in literature and found that the average age of onset of HH was 2.49 years [5]. Among 214 HH patients reported by Beijing Tiantan Hospital, only 2.3% developed HH in adulthood [7]. In the present study, we retrospectively review the clinical characteristics of an adult HH patient without GS, who was treated in the Department of Neurology of our hospital. We will also provide a review of previous studies on adult HH.

## Case presentation

### Clinical Data

**Fig. 1:**
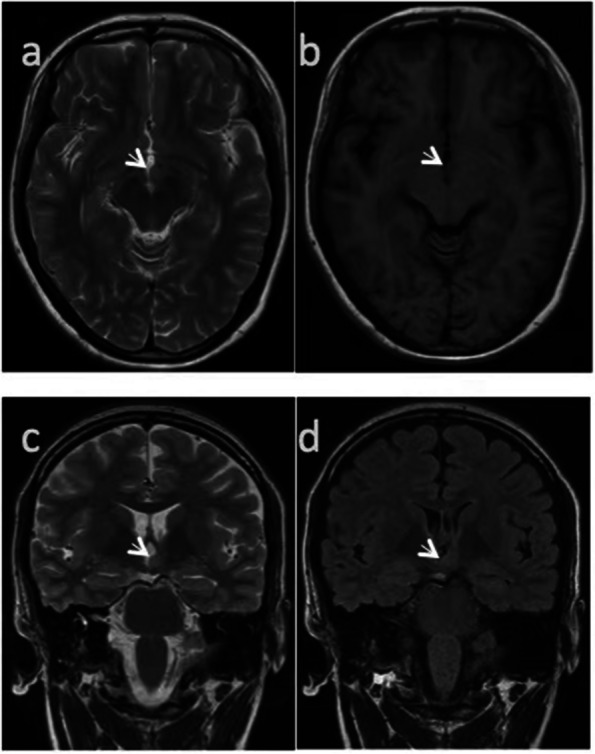
Cranial magnetic resonance imaging (MRI). Axial T2WI (**a**), T1WI (**b**), coronal T2WI (**c**), and T2 FLAIR (**d**) all showed presence of HH in the left side, with equal signal shadows. The HH had a size of 9 mm * 5 mm, and protruded into the third ventricle

**Fig. 2:**
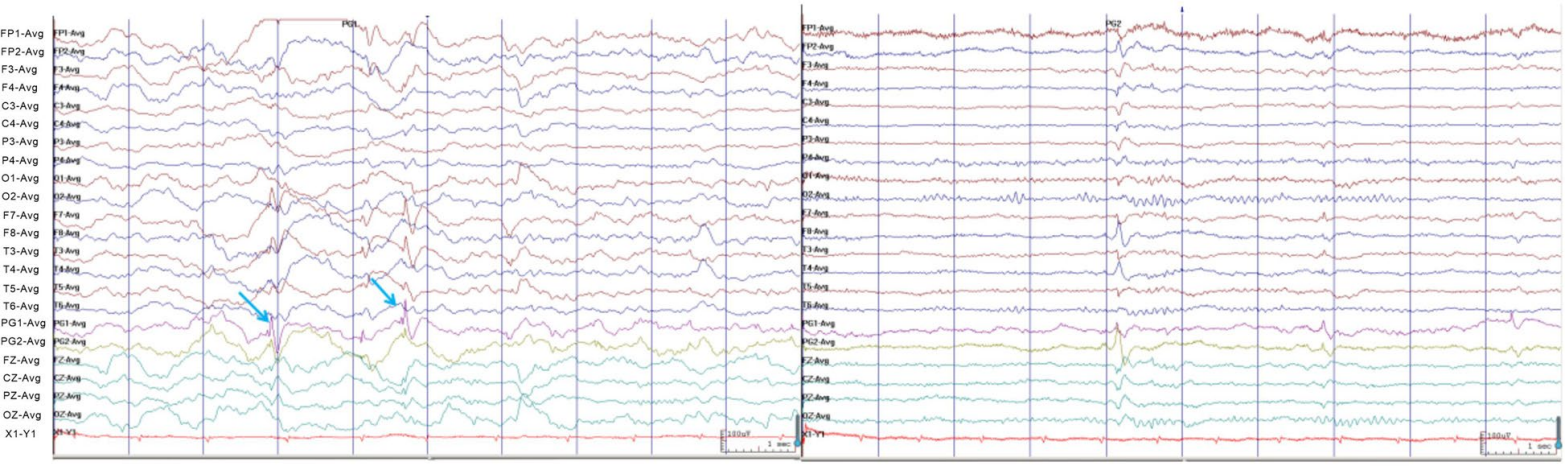
EEG in the interictal period, indicating abnormal discharges in the bilateral temporal areas and sphenoid electrode area(blue arrow), significant on the left side

**Fig. 3:**
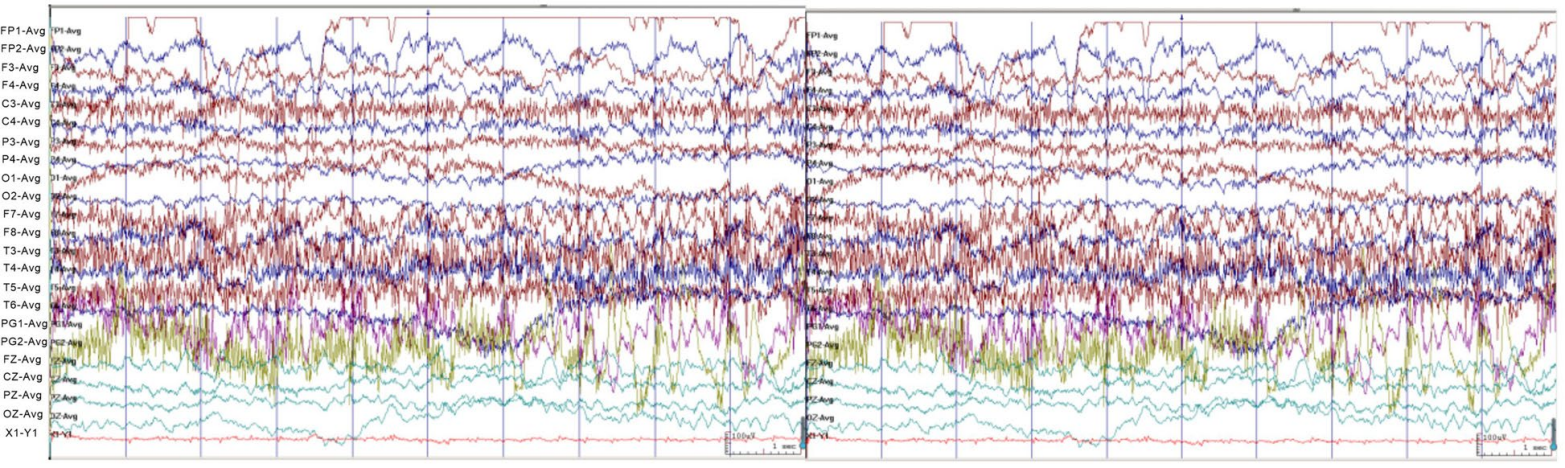
EEG recordings during a seizure. The EEG traces showed fast-wave activity in bilateral temporal areas, significant on the left, and then evolved into sharp-wave rhythms in the left temporal area, frontal pole, and frontal area, and finally spread to the whole lead, showing slow-wave rhythm distribution

A 24-year-old female patient was admitted to our hospital for "paroxysmal loss of consciousness over more than 1 year". In April 2020, the patient developed episodic unconsciousness, first deja vu, fear, and palpitations, followed by unconsciousness accompanied by vocalization, lip smacking, straightening of the left upper limb, involuntary groping of the right hand, and wandering around, which lasted for 2–3 minutes each time, and occurred 2–3 times a week. She had received treatment at other hospitals and had been diagnosed with "neurosis". The patient’s condition did not improve after treatment with "Deanxit and escitalopram oxalate". In mid-March 2021, a new type of seizure appeared, manifested as a dazed condition, slanting of the right corner of the mouth, loss of consciousness, and uplifting of the right upper limb. Thereafter, the head, neck, and body deflected to the right, and she fell to the ground slowly, with the upper limbs being straight and rigid for half a minute. The seizure attacks occurred three times a week on average. She was admitted to our hospital on April 25, 2021. The neurological examination showed no obvious abnormality. Cranial magnetic resonance imaging (MRI) showed a HH on the left (Fig. 1). Video electroencephalogram (EEG) monitoring showed abnormal waveforms during interictal periods (Fig. 2). During the monitoring period, she had one seizure attack, characterized by accelerated heart rate, lifting and stiffening of the right upper limb, loss of consciousness, lip smacking, involuntary groping with the left upper limb, and a pedal-like movement of the right lower limb. EEG manifestations during this seizure are presented in Fig. 3.

**Fig. 4:**
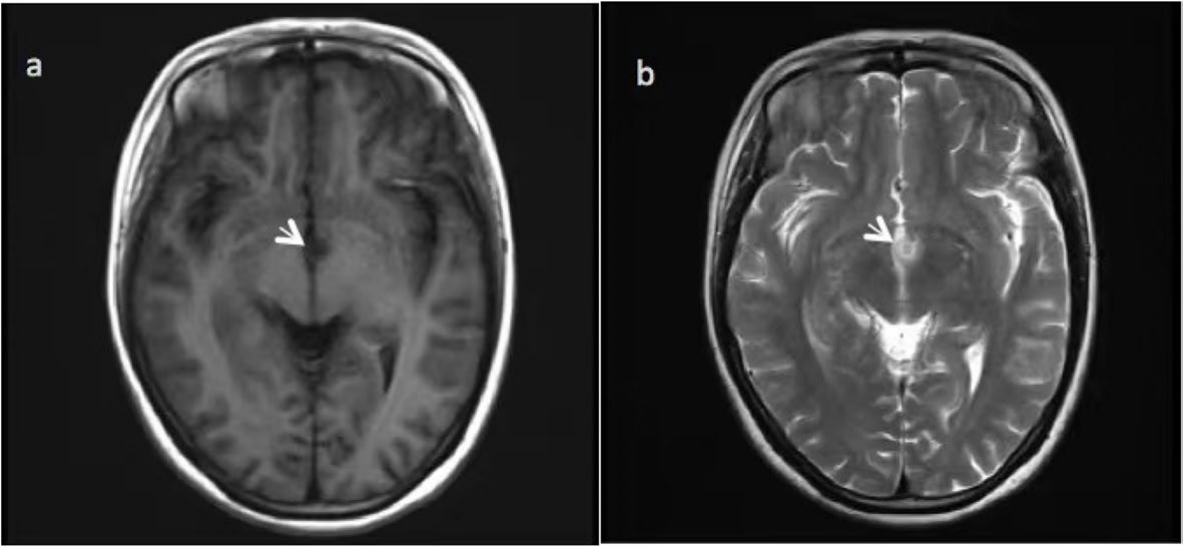
Postoperative cranial MRI T1WI (**a**) and T2WI (**b**)

After discussion with the clinical team, the patient’s diagnosis was confirmed to be symptomatic epilepsy and HH. The primary seizures were focal with disturbances of consciousness, leading to secondary generalized seizures. In May 2021, the patient was treated with stereotactic radiofrequency thermocoagulation (SRT) at a maximum temperature of 60–65 °C for 60 s, followed by treatment with 1 g of levetiracetam twice per day and 300 mg of oxcarbazepine twice per day after the operation. The patient experienced no seizures during the year after the surgery. In June 2022, the patient stopped taking the drug on her own, and no seizures were observed until October 2022. The postoperative cranial MRI is shown in Fig. 4.

## Discussion

The case presented in our report experienced a variety of seizure forms that appeared in adulthood, manifested mainly as focal seizures accompanied by disturbance of consciousness and secondary generalized seizures. The patient had no history of GS. This is rarely seen as most HH patients have epilepsy in the form of GS or have a history of GS [8, 9]. A recent study reported that most HH patients without GS would have a very late-onset age [10]. Sone et al. [11] also reported a female case of adult-onset HH with a disease course of eight years, presenting as intractable epilepsy without GS. In our case, clinical manifestations and EEG examination suggested that the seizures may originate from the temporal lobe. However, brain MRI examination revealed a left-sided HH with a diameter of 9 mm, and no abnormal signals were observed in the left temporal lobe.

A similarity between the case reported by Sone et al. and our case is that both cases showed clinical manifestations and video EEG activity that suggested origination of the seizures from the temporal lobe. To clarify the origination of seizures, Sone et al. [11] using stereotactic intracranial EEG (SEEG) recordings and found that the ictal discharges from the HH preceded those from other regions including the left hippocampus. In our case, although the seizure was not confirmed to originate from the HH by SEEG, it was effectively controlled after destruction of the HH by SRT. So why did these patients show symptoms similar to temporal lobe seizures? Ictal single-photon emission computerized tomography studies have demonstrated the presence of increased functional connectivity (FC) between the HH/hypothalamic region and the ipsilateral thalamus, especially with the anterior thalamic nucleus (a relay to the anterior cingulate and limbic circuit) and the thalamic nucleus (a relay to the orbitofrontal, anterior cingulate, and medial premotor, as well as the parietal cortical areas) [12–14]. HH is thus able to form a complex epileptogenic network over the range of the HH through various anatomical and functional connections [10, 15]. Kahane et al. [16] reported the complete Grenoble series of 5 cases showing gelastic or dacrystic seizures correlated with discharges in the HH, although other types of seizures were related to discharges affecting the neocortical regions. The HH might work as a pacemaker, and the seizure activities of the HH are generated by the intrinsic pacemaker neurons [17]. The seizure activity can spread to adjacent cortical structures such as the frontal and temporal lobes, which then act as secondary foci of epileptogenesis [18]. Two major phenotypes can be distinguished based on electroclinical features: (i) focal seizures with epigastric or dejà-vu auras, loss of consciousness and oroalimentary or gestural automatisms suggestive of temporal lobe involvement; and (ii) motor seizures with tonic, atonic, myoclonic, or versive phenomena, suggesting frontoparietal network involvement [19]. Thus, in our patient, the seizures of similar temporal origin were caused by HH triggering of temporal neocortical discharges.

The differences between the two cases lie in the course and prognosis. The patient reported by Sone et al. had a disease course of eight years, and took medications of "sodium valproate, carbamazepine and lamotrigine", but still experienced complex partial seizures approximately once every 2-3 months. The patient was then treated with SRT, and only took lamotrigine after the operation. Although the seizures did not recur after three years of follow-up, the patient’s cognitive function did not recover. In our report, the case had a disease course of one year, with seizures being effectively controlled in the early stage of the disease without cognitive impairment. The seizure activities of the HH are usually present as drug-resistant epilepsy, but as the epilepsy intrinsically arises within the hamartoma, ablation of the hamartoma by any method can result in remission of the seizure activity [13, 20, 21]. Schulze-Bonhage et al. [22] also found that early discovery, diagnosis, and treatment offer better control of epileptic symptoms. Treatment at the late stages is associated with a lower success rate and a higher incidence of complications such as cognitive impairment. A longer duration of epilepsy is also been related to the poorer prognosis after HH-targeted surgical procedures. In the Strasbourg-Kork series, 80% of patients with epilepsy duration less than 10 years achieved complete seizure freedom, whereas only 20% of those with disease for over 20 years achieved seizure-free [23]. These results indicate that early diagnosis and early treatment are of great significance in improving the prognosis of HH patients.

## Conclusions

In conclusion, HH without gelastic seizures is easily misdiagnosed as it may occur in a form similar to temporal lobe seizures. Early treatment of HH leads to improved control of epileptic symptoms and patients with a longer course of disease may have more complications. Therefore, early diagnosis and treatment is of great significance to improve the prognosis of HH patients.

## Data Availability

Not applicable.

## Abbreviations

EEG: Electroencephalogram
GS: Gelastic seizure
HH: Hypothalamic hamartoma
MRI: Magnetic Resonance Imaging
PP: Precocious puberty
SEEG: Stereotactic intracranial Electroencephalogram
SRT: Stereotactic Radiofrequency Thermocoagulation
